# Clinical Importance of CDKN2A Loss and Monosomy 10 in Pilocytic Astrocytoma

**DOI:** 10.7759/cureus.4726

**Published:** 2019-05-23

**Authors:** Daniel N Cagney, Michael B Miller, Adrian Dubuc, Ivana Delalle, Azra H Ligon, Ugonma Chukwueke, Ossama Al-Mefty, Ayal Aizer, Keith Ligon, Patrick Wen

**Affiliations:** 1 Radiation Oncology, Dana-Farber / Brigham and Women’s Cancer Center, Harvard Medical School, Boston, USA; 2 Pathology, Dana-Farber / Brigham and Women’s Cancer Center, Harvard Medical School, Boston, USA; 3 Neuro-Oncology, Dana-Farber / Brigham and Women’s Cancer Center, Harvard Medical School, Boston, USA; 4 Neurosurgery, Dana-Farber / Brigham and Women’s Cancer Center, Harvard Medical School, Boston, USA

**Keywords:** pilocytic astrocytoma, pilocytic astrocytoma, low grade glioma, low grade glioma, malignant transformation, malignant transformation, cdkn2a loss, pten loss, cdkn2a loss

## Abstract

This case of a radiation-naive patient with pilocytic astrocytoma highlights how deletions of CDKN2A (cyclin-dependent kinase Inhibitor 2A) and PTEN (phosphatase and tensin homolog) portended a poor clinical outcome. Pilocytic astrocytomas are grade 1 tumors usually occurring in children and young adults with KIAA1549-BRAF fusion defining the majority of pilocytic astrocytomas. The presence of CDKN2A and PTEN loss may be associated with aggressive biology in pilocytic astrocytoma and further studies should include comprehensive genomics in a larger series of adult pilocytic astrocytoma to evaluate this previously unreported finding. Providers need to be aware of this possibility given the potential for poor outcomes.

## Introduction

Pilocytic astrocytomas are grade 1 tumors usually occurring in children and young adults and are typically associated with an excellent prognosis [1]. KIAA1549-BRAF fusion defines the majority of pilocytic astrocytomas, most often representing the sole genomic aberration [2, 3]. While chromosomal aneuploidy has been identified in adults with pilocytic astrocytoma, only a few cases have reported deletions of the tumor suppressor genes CDKN2A (cyclin-dependent kinase Inhibitor 2A) and PTEN (Phosphatase and tensin homolog), which are associated with high-grade gliomas and are very rare in low-grade gliomas, including pilocytic astrocytomas [4-6]. In the present report, we describe the aggressive clinical course of a patient with pilocytic astrocytoma harboring canonical KIAA-BRAF fusion. Notably, at the time of diagnosis this patient’s tumor also showed concomitant homozygous CDKN2A deletion and monosomy 10.

## Case presentation

**Figure 1:**
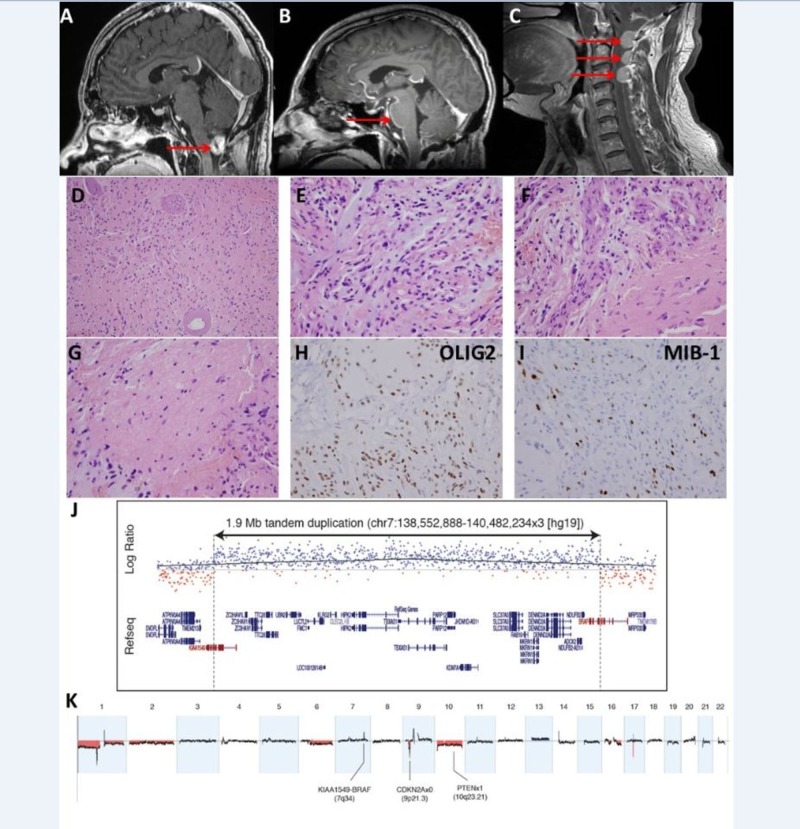
Pilocytic astrocytoma with malignant course. (A-C) imaging, (D-I) histopathologic findings, (J, K) chromosomal microarray. (A) Preoperative sagittal T1 contrast-enhanced brain MRI revealing enhancing 1.5 cm posterior fossa mass. (B) Sagittal T1 contrast-enhanced brain MRI revealing local recurrence in the cerebellar cavity and leptomeningeal enhancement along the anterior aspect of brainstem and cervical spinal cord. (C) Sagittal T1 contrast-enhanced cervical spine MRI showing dural recurrence in the superior aspect of cervical spine. (D) Glial tumor cells in haphazard arrangement with thickened and hyalinized blood vessels (medium-power, 200x magnification). (E) Tumor cells with myxoid background (high power, 400x magnification). (F) Biphasic tumor histologic appearance, with proliferated blood vessels (400x). (G) Tumor stroma including eosinophilic structures resembling Rosenthal fibers. (H) OLIG2 immunohistochemistry shows the majority of cells to be positive. (I) MIB-1 highlights proliferation index which was quantified at 11%. (J) Focused view of copy number gain involving BRAF (7q34). (K) Genome-wide view illustrating multiple copy number aberrations, including nullisomy for CDKN2A and PTEN loss (occurring via monosomy 10).

A 53-year-old gentleman presented in early 2015 with sudden onset of left-sided hearing loss. MRI brain revealed a 1.5-cm cerebellar lesion (Figure 1A). He underwent a suboccipital craniotomy and gross total resection. Histopathology was consistent with pilocytic astrocytoma, grade I/IV (Figure 1D-1I). Chromosomal microarray hybridization cytogenetic testing revealed multiple aberrations (Figure 1J-1K), including x single copy gain at 7q34 with breakpoints in BRAF and KIAA1549, consistent with a tandem duplication leading to the BRAF-KIAA1549 fusion that is observed in pilocytic astrocytomas (confirmed by targeted exome sequencing). However, cytogenetics also revealed one to two copy loss involving CDKN2A and monosomy 10, producing a single copy loss of PTEN. No adjuvant treatment was recommended. He represented in January 2016 with increasing headaches and visual changes. Staging MRI brain/whole spine revealed new enhancing lesion in the surgical cavity, multiple enhancing dural-based lesion in the cervical spine and new diffuse leptomeningeal enhancement (Figure 1B-1C). His case was discussed at our neurooncology multidisciplinary conference with the recommendation to proceed with craniospinal irradiation (CSI). Unfortunately, three days following initiation of CSI, he acutely deteriorated and ultimately succumbed to his disease.

## Discussion

Pilocytic astrocytomas are typically low-grade tumors with slow growth and favorable prognosis, with 10-year survival rates of >95% following surgical resection alone. The majority of pilocytic astrocytomas, including the case reported here, occur in the cerebellum and contain a tandem duplication of BRAF with the adjacent gene KIAA1549, resulting in a BRAF fusion gene with constitutive kinase activity [1]. BRAF fusion gene is thought to be the oncogenic driver, as it typically is the sole significant genetic aberration identified [2, 3]. With the common mechanism of MAP (mitogen-activated protein) kinase pathway activation, oncogenic drivers found in other cases of pilocytic astrocytoma include BRAF V600E mutation, NF1 (Neurofibromatosis type 1) biallelic inactivation, FGFR1 (Fibroblast growth factor receptor 1) mutation or duplication, and NTRK (Neurotrophic tyrosine kinase) duplication [3].

Tumors carrying the histologic diagnosis of pilocytic astrocytoma only very rarely demonstrate high-grade histologic features or malignant behavior. The proportion of pilocytic astrocytomas with concerning histologic features, such as ≥4 mitotic figures in 10 high power microscopic fields, hypercellularity, and nuclear atypia, has been found to be 0.9-1.7% [4,5]. One series of such tumors reported a correlation between "anaplastic features" and aggressive behavior [5].

A study of 886 low-grade gliomas bearing the canonical KIAA1549-BRAF fusion gene of pilocytic astrocytoma found no patients with anaplastic transformation leading to disease-related death [6]. This same study also described 26 cases of apparent pilocytic astrocytoma that progressed to high-grade glioma, with none of this group bearing the KIAA1549-BRAF fusion. Instead, many of these progressing pilocytic astrocytomas showed BRAF V600E mutation along with CDKN2A deletion, a finding that suggests that certain additional aberrations can produce aggressive behavior in tumors driven by excessive BRAF signaling. It is also worth noting that most previous reports of high-grade pilocytic astrocytomas involved recurrent tumors following irradiation of an initial low-grade pilocytic astrocytoma. Therefore, features of the post-radiation lesion may not represent the initial biology of the tumor, and may have different biology than the radiation-naive tumor we report here.

Another study examined 92 pilocytic astrocytomas of varying histology [7]. Among the 25 cases which showed "anaplastic" histology, 32% showed PTEN loss, and 20% showed CDKN2A loss, changes which were not observed in pilocytic astrocytomas with conventional histology. Furthermore, three cases showed both CDKN2A loss and PTEN loss, as seen in the case we report here. Given the tendency for such tumors to show aggressive behavior, these genetic alterations may be responsible for increased cell proliferation. While two of these three cases did not have BRAF duplication, one case did bear BRAF duplication, and therefore displayed all three of the principal genetic alterations discussed in the case we report here. However, that case also involved a history of radiation, a distinct and unfavorable event not found in our patient.

## Conclusions

Here we report a unique case of BRAF-duplicated pilocytic astrocytoma which demonstrates loss of CDKN2A and PTEN in the initial tumor, a previously unreported finding. This case suggests that, even in radiation-naive pilocytic astrocytoma, CDKN2A and PTEN loss may produce aggressive biology. Providers need to be aware of this possibility given the poor outcomes associated with this clinical scenario.
